# Superior Semicircular Canal Ampullae Dehiscence As Part of the Spectrum of the Third Window Abnormalities: A Case Study

**DOI:** 10.3389/fneur.2017.00683

**Published:** 2017-12-19

**Authors:** Eugen Constant Ionescu, Nasser Al Tamami, Alexandra Neagu, Aicha Ltaief-Boudrigua, Stephane Gallego, Ruben Hermann, Eric Truy, Hung Thai-Van

**Affiliations:** ^1^Service Audiologie et Explorations Otoneurologiques, Hospices Civils de Lyon, Centre Hospitalo-Universitaire Lyon, Lyon, France; ^2^Service d’Imagerie Médicale et Interventionnelle, Hospices Civils de Lyon, Centre Hospitalo-Universitaire Lyon, Lyon, France; ^3^Institut des Sciences et Techniques de la Réadaptation, Université Claude Bernard Lyon 1, Lyon, France; ^4^Service ORL et Chirurgie Cervico-Faciale, Hospices Civils de Lyon, Centre Hospitalo-Universitaire Lyon, Lyon, France; ^5^Lyon Neuroscience Research Center, IMPACT Team, CRNL, INSERM U1028, CNRS UMR5292, Lyon, France; ^6^Université Claude Bernard Lyon 1, Lyon, France

**Keywords:** ampullar dehiscence, tullio phenomenon, sound-induced falls, third window syndrome, superior canal dehiscence syndrome

## Abstract

A 60-year-old man was referred to the ENT department for intense episodic vertigo triggered by loud sounds. Pure tone audiometry and otoneurological assessment, including videonystagmography using auditory stimulation and cervical vestibular evoked myogenic potential measures, conducted to the hypothesis of a third window syndrome in the left ear. Results from the high-resolution computed tomography of the petrous bone confirmed the hypothesis and revealed the presence of a submillimeter semicircular canal dehiscence, located between the left lateral and superior semicircular canal ampullae on the left side.

## Background

The third window abnormalities are disorders affecting the bony labyrinthine structures (1). Pathophysiological mechanisms underlying these abnormalities could be explained as following: (1) in air conduction, a portion of the energy conducted by the perilymph is shunted away from the cochlear partition resulting in a conductive hearing loss (CHL). Furthermore, the pressure difference between the two sides of the cochlear partition is increased resulting in an improvement of the cochlear response to the bone conduction. (2) The shunted utriculopetally energy through the labyrinth make the vestibular organs sensitive to sound (pressure) stimulation. This is known as Tullio phenomenon (TP) (2). The superior semicircular canal dehiscence (SSCDS) is the most documented among these lesions (3–9). The SSCDS’ symptoms refer to autophony, CHL, and TP (3). Following Ewald’s laws, the ampullar nerve is, respectively, inhibited or stimulated by loud sounds, variations of the middle ear or intracranial pressure, resulting in a specific nystagmus. Grieser (4) modeled the interaction between the vestibular fluids and the membranous labyrinth confirming this SSCDS theory. He demonstrates how stapes-generated oscillations of the perilymph flux are compensated by the membranous semicircular canal contractions, which then deflect to the superior ampullar crista.

Ho et al. (5) described similar clinical conditions and proposed the broader term of “third window abnormalities spectrum.” As hypothesized by Merchant (1) a number of disparate disorders that produce hearing loss or vertigo would be explained by a third-window mechanism. Recently, Gubbels et al. (10) reported a case of a dehiscence of the posterior semicircular canal which included the ampullar region in relation to a high jugular bulb position. Symptoms were similar to those generated by the SSCDS in relation with the superior petrous sinus (6). In the present article, we report a case with ampullar dehiscence where the clinical picture was not in line with the SSCDS.

## Case History

A 60-year-old male was referred to our department by an ENT specialist. The patient was working in the metallurgy field and, since recently, he started to experience vertigo when exposed to loud noise at work, provoking frequent falls. In the ENT report, it was mentioned that the patient did not have traumatic or inflammatory otological history and any auditory symptoms or loss of consciousness. Blowing nostrils conducted to a slight vertigo, without any falls. The specialist concluded to normal otoscopic examination and to a sensorineural hearing loss. He added that during the audiometry, the patient experienced dizziness when being tested in the left ear. The physician suspected then SSCDS and asked for high-resolution computed tomography (HRCT) to investigate the petrous bone. The radiologist ruled out a third window abnormality. As vestibular symptoms persisted, the patient was referred to our clinic for further investigations.

## Procedure

In our center, the patient underwent ENT examination, auditory, vestibular tests, and a HRCT of the petrous bone.

### ENT Examination and Auditory Tests

Otoscopic examination was performed with an OPMI^®^ Sensera, Zeiss microscope. Hearing sensitivity was measured with a Madsen Astera—Otometrics audiometer. Acoustic impedancemetry and middle ear reflexes were done with a Madsen Zodiac 901 tympanometer.

### Vestibular Tests

Standard Videonystagmosgraphic (VNG) (Ulmer System^®^ from Synapsis SA) protocol included: the head shaking test, the fistula test, and Valsalva’s manoeuver against a closed glottis as decribed by Hain (11).

Video Head Impulse Test (VHIT) was performed with ICS—Impulse Test Otometrics^®^ system. Cervical vestibular evoked myogenic potentiels (cVEMPs) were done with a Bio-Logic^®^ Nav-Pro system in air conduction with 750 Hz tone bursts.

### Radiologic Imaging

The CT scanner used in this case study was a scanner GE GSI Revolution, USA. Slices were acquired helically in the axial plane at nominal 0.625 mm slice thickness with a 50% overlap of 0.312 mm. All images were obtained in ultrahigh resolution at 140 kV and 200 mAs/section. The primary images were retargeted in the axial and coronal planes of the lateral semicircular canal, to a 60 mm field of view at a 512 matrix for an isometric voxel. The retargeted axial scans were then reformatted in the plane of Pöschl (parallel to the plane of the superior semicircular canal), using “AWserver software,” GE Healthcare.

The reformatted 0.2 mm slices were non-overlapped, and scans were viewing at a window level of 800 HU and width of 4,000 HU.

## Results

The main findings can be summarized as follows.

**Figure 1:**
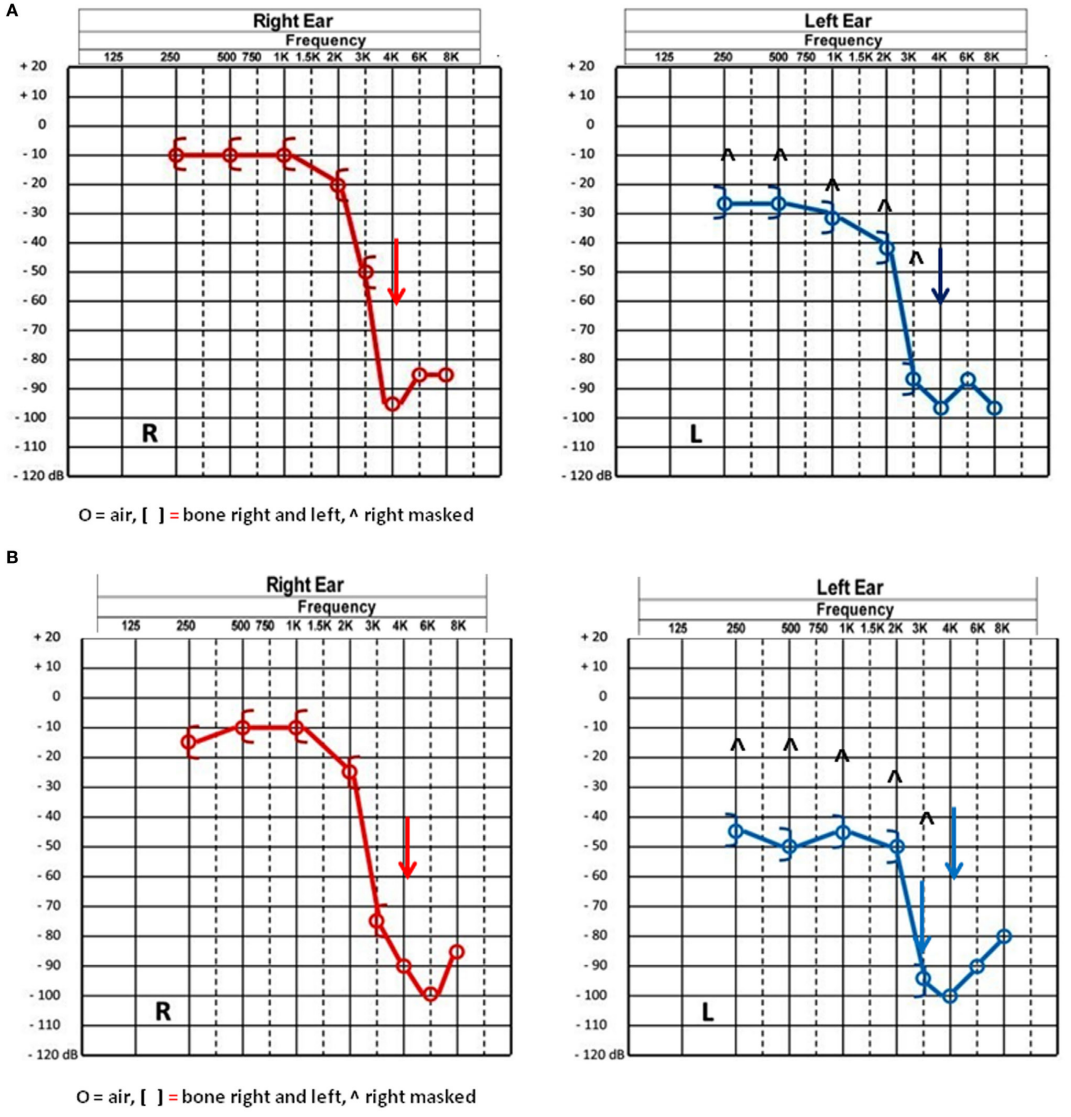
**(A)** Pure tone audiometry results before surgery showing a bilateral characteristic noise induced neurosensorial hearing loss on high frequencies. **(B)** Pure tone audiometry postsurgery results showing a 15–20 dB decrease in the left ear thresholds from 250 to 1,000 Hz and in the right ear at 3,000 and 6,000 Hz.

**Figure 2:**
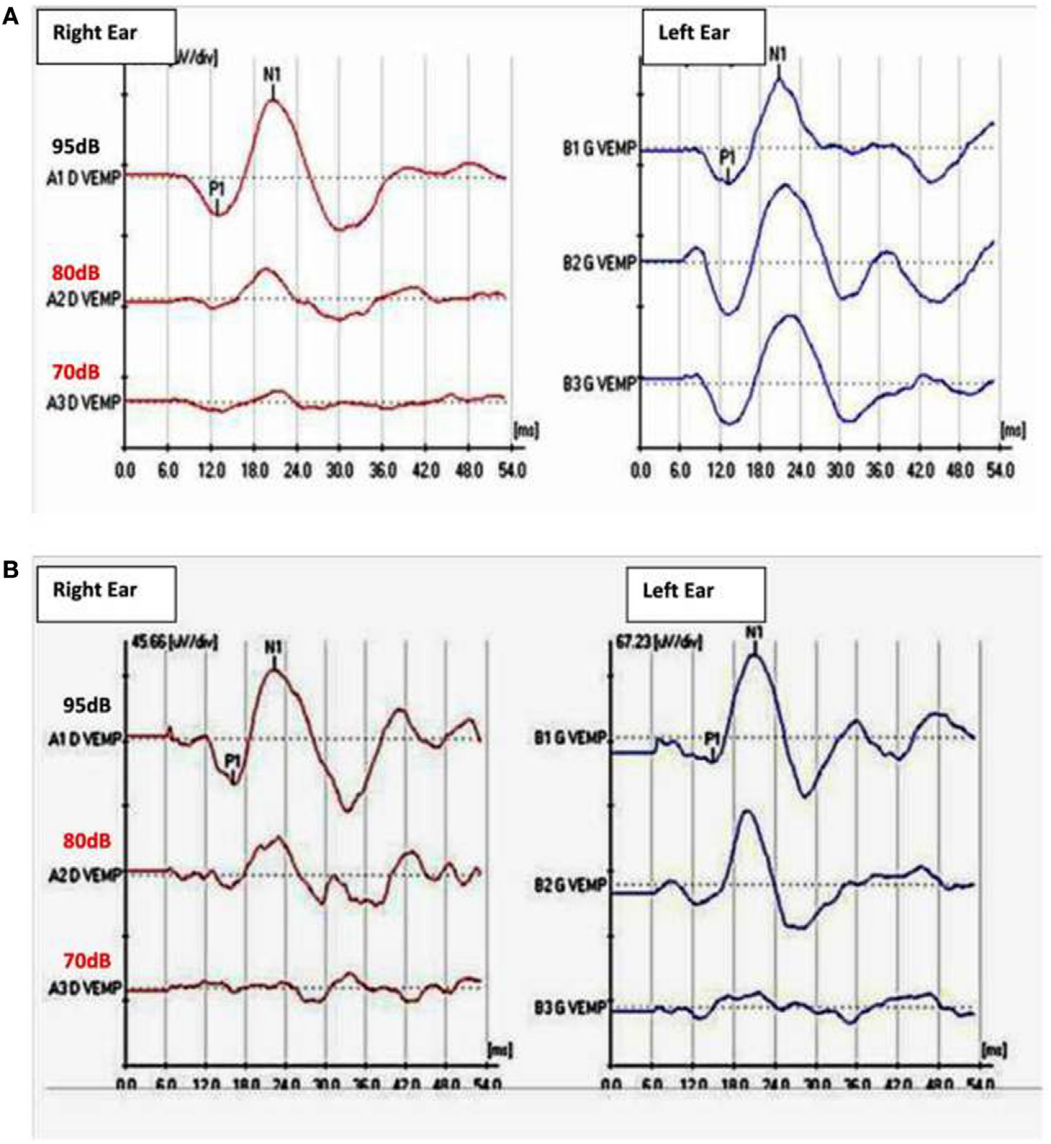
**(A)** Cervical vestibular-evoked myogenic potentiels (cVEMPs) before surgery with abnormal low threshold at 70 dBnHL in the left ear with a 750 Hz tone burst stimulation. Normal threshold VEMPs in the fright ear at 95 dBHL. **(B)** cVEMPs after surgery: threshold increased to 80 dBnHL on the left side. Normal threshold at 95 dBnHL on the right ear.

Normal tympanic membranes and normal tympanometry were obtained. Ipsi and contralateral stapedial reflexes were normal in both ears. PTA results showed a mild to severe sensorineural hearing loss in both ears (Figure 1A). A slight vertigo without nystagmus was evoked by Valsalva’s maneuver. VNG subtests and VHIT results were normal. cVEMPs results showed an abnormal low threshold at 70 dBnHL on the left side (Figure 2A). Radiological imaging results revealed that the HRCT of the petrous bone was normal.

## Further Investigations

Since the radiological findings were reported as being normal a surgical exploration for perylimphatic fistula suspicion was decided. An endaural approach was then used and a blocking of both oval and round windows was performed with abdominal fat and fibrin glue (12). There were no complications following the surgical procedure and the patient was discharged 24 h after the surgery. Three weeks later, PTA control showed a slight decrease for thresholds at 250–1,000 Hz in the left ear (Figure 1B). Ipsilateral cVEMPs were present at 80 dBnHL (Figure 2B).

Unfortunately, symptoms remained unchanged for the patient. Sound-induced vertigo occurred exactly in the same conditions as those in presurgery. An otolith form of TP as described by Dieterich et al. (13) was then stated as a hypothesis. A left ear plug protection was prescribed to the patient. By using this protection, providing an auditory attenuation up to 35 dB (Quies foam earplugs^®^), symptoms stopped and the patient returned to work.

**Figure 3:**
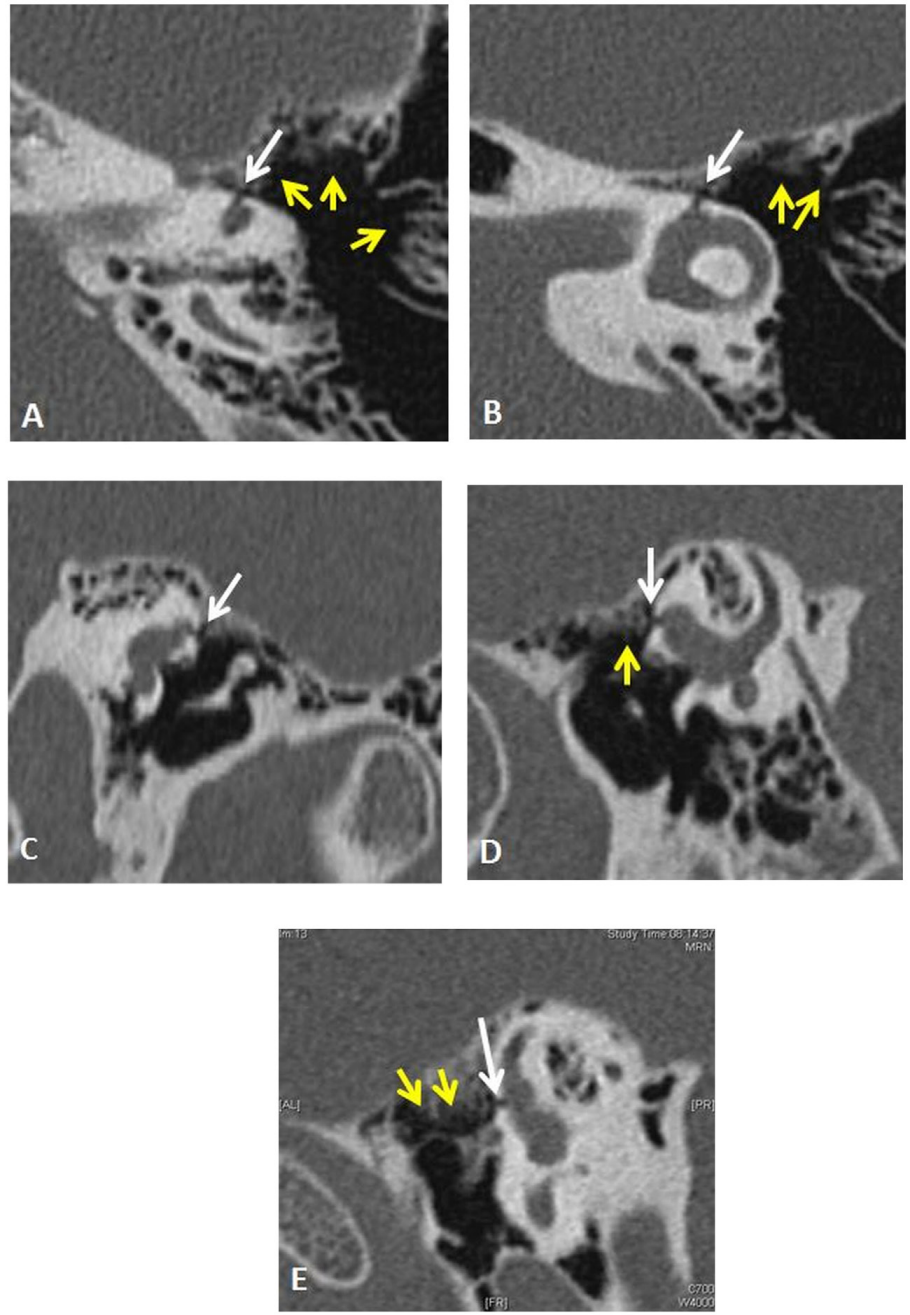
Left temporal bone high-resolution computed tomography (HRCT) demonstrates by its shape and topography (white arrows) an original ampullar dehiscence of the superior semicircular canal. The infra-millimetric (~0.5 mm) and tubular in shape dehiscence was disposed between the ampullaes of the lateral and superior semicircular canal and the medial epitympanic wall. An important pneumatization of perilabyrinthine air cells was observed [yellow arrows **(A,B,D,E)**]. **(A,B)** axial left temporal CT scan. **(C)** Reformatted image of the left labyrinth (oblique coronal). **(D)** Reformatted image of the left labyrinth (oblique sagittal). **(E)** Reformatted image of the left labyrinth (oblique sagittal). The ampullar dehiscence on the left superior semicircular canal’s anterior crus was visible on two perpendicular planes [axial **(A,B)**, coronal plane **(C)**, Pöschl plane **(D)**, and in oblique sagittal plane **(E)**]. Its diameter was measured between 0.4 and 0.5 mm.

Although the patient was alleviated, 6 months later during the follow-up consultation, he reported having communication difficulties and was concerned also about his security at work. Additional neurovestibular tests were decided in auditory condition which triggers the TP. Videonystagmography was performed with the Digital Nystagview^®^, Synapsis system. The patient was seated and wore goggles and pure tones at 500, 750, 1,000, 2,000, 3,000, 4,000, and 6,000 Hz were presented under supra-aural earphones at level of 80 dBnHL and increased by 10 dB steps in both ears. An intense downbeating, slightly clockwise torsional nystagmus was obtained at 120 dBnHL at 2,000, 3,000, and 4,000 Hz when the left ear was stimulated (Video S1 in Supplementary Material). Nystagmus and vertiginous sensations were maximal at 2,000 and 4,000 Hz. These findings pointed toward stimulation by sound of the left superior ampullary nerve. Therefore, the attention was focused on ampullar regions when revisiting the HRCT. The retargeted scans were then reconstructed by the radiologist as recommended (7) in axial, coronal, and oblique sagittal planes paralleling to the superior semicircular canal. He reported that the thickness of the six semicircular canals and of the otic capsule, the vestibular and the cochlear aqueducts dimensions were all normal, ruling out the suspicion of third window abnormalities at any of these levels. However, the specialist identified a left superior ampullar dehiscence of unknown origin, with a dimension inferior to 1 mm (Figures 3A–E).

A surgical solution was a treatment option since an obstruction of the ampullar dehiscence by a smooth drilling of the left superior ampullar region appeared technically possible with minimal risks. Amplification with hearing aids was also discussed with the patient. The objective was to compensate the patient’s hearing loss for sounds under 70 dB SPL while electronically limiting output levels exceeding 80 dB SPL on the left ear especially between 2,000 and 4,000 Hz where vestibular effects were present. The patient chose the amplification option and both ears were fitted. At the control visit, 6 months later, he was asymptomatic and his auditory comfort was considerably improved.

## Discussion

This 60-year-old man reporting severe episodic TP had an atypical clinical picture of SSCDS: auditory investigations did not show a CHL and the vestibular tests results showed a nystagmus in a direction opposed to the expected side when the patient was hearing loud sounds (usually in SSCDS the nystagmus direction is with upward torsional slow phases) (8). This is in concordance with Kaski et al. (2). which emphasized that SSCDS is not always the underlying cause for TP. Gianoli and Soileau study (9) revealed similar cases with audiovestibular findings suggesting a third window dysfunction and negative HRCT. They postulated that changes in the intracranial pressure may cause compliance perturbation at both windows’ regions, resulting in a potential middle ear perilymphatic fistula. Our initial decision to perform a surgical exploration of the inner ear’s windows region was based on the same postulate. However, our assessment was refined and test results conducted toward the identification of a superior semicircular canal ampullae dehiscence.

Two scenarios are possible to explain the mechanism of this particular dehiscence. One is similar to the one associated to SSCDS. Triggered by stapes vibrations, a perilymphatic flow is oriented from the oval window to the third window situated on the ampulla of the superior semicircular canal. The dissipated auditory energy would be lower than in SSCDS since the distance between the stapes and the superior canal ampulla is shorter than the distance to the convex superior canal to the stapes. Thus, there is no significant CHL associated to the TP. This hypothesis is supported by the existence of a lower cVEMPs threshold which indicates that a perilymphatic flow is shunted through vestibular structures (first slide in Presentation S1 in Supplementary Material). On the other hand, the Grieser’s model (4) predicts that in SSCDS, the greatest nystagmus should be observed when auditory stimulus is around to 1,000 Hz, with no significant nystagmus at higher frequencies. However, this model does not apply to the present case report, as an unexpected prompt vestibular reaction was observed with higher frequencies stimulations.

The second hypothesis explains TP in a similar way as to vertigo in patients after fenestration surgery (14, 15) or in case of idiopathic dehiscence of the lateral semicircular canal (16). The sound waves through the ampullar dehiscence generate a perylimphatic flow transmitted directly to the membranous superior canal which then stimulates ampullar crista (second slide in Presentation S1 in Supplementary Material). The propagation of the sound between the external auditory canal and the dehiscence would be facilitated by an important pneumatization of the temporal bone. This second hypothesis is supported by the persistence of the symptoms despites the surgical blocking of both oval and round windows, although the threshold of the left cVEMP decreased in post-surgery measures.

## Concluding Remarks

Clinical features of a patient with a submillimeter results superior semicircular canal ampullar dehiscence are reported. Nystagmus and cVEMPs results were the major findings leading to the hypothesis of a variant of the third window syndrome. Although the mechanism underlying the stimulation of the perilymph remains uncertain, the observed nystagmus in this patient was undoubtedly generated by an utriculofugal flow as shown in the Presentation S1 in Supplementary Material. Further experimental studies are needed before validating one of the proposed mechanisms mentioned above. However, there are indications that the second scenario is the most likely hypothesis to be confirmed. Those are the absence of a CHL and nystagmus with the Valsalva maneuver (1, 3–9) and the unusual sound-induced vertigo leading to falls. Furthermore, the sound frequencies inducing vertigo were higher than those predicted by the Grisers’ model for the SSCDS. Thus this mechanism appeared to be closer to the principles of fenestration of the lateral semicircular canal type surgery proposed by Lempert (15) for severe otosclerosis which may generate TP (17). Although this is the first case report on superior semicircular canal ampullar dehiscence, clinicians should be aware about this pathology which, theoretically, may occur over other ampullar regions. In patients with intense TP, no CHL and no evidence of “classic” semicircular canal dehiscence, the temporal bone HRCT should include a careful exploration of the ampullar regions with minimally slices of 0.625 mm (7) or less at 0.24 mm (9).

## Ethics Statement

This study/case report was carried out in accordance with the recommendations of Haute Autorité de Santé (https://www.has-sante.fr/portail/jcms/c_2036961/en/best-practice-guidelines), France. A written informed consent was obtained from the patient.

## Author Contributions

EI—observed and diagnosed the patient, he elaborated the pathophysiological hypothesis. NT—second position—he elaborated the text and putted in form different exams, verifying the written English quality. AN—elaborated with the first author the video nystagmography protocole while stimulating in earphone left ear’s patient. AL-B—the team’s radiologist performed demanded CT scan examination leading to the positive diagnosis. SG—the audiologist which fitted patient’s ear protection and the hearing-aid finally. RH—he performed with the first author the first vestibular assessment: VNS and cVEMP. ET—the surgeon of the team, he performed the middle ear windows blockage. HT-V—contribution in the elaboration of the theory concerning the Tullio’s phenomenon observed in this case report.

## Conflict of Interest Statement

The authors declare that the research was conducted in the absence of any commercial or financial relationships that could be construed as a potential conflict of interest.

## References

[1] Merchant SN Rosowski JJ. Conductive hearing loss caused by third-window lesions of the inner ear. Otol Neurotol (2008) 29(3):282–9. 10.1097/mao.0b013e318161ab24 18223508 PMC2577191

[2] Kaski D Davies R Luxon L Bronstein AM Rudge P. The Tullio phenomenon: a neurologically neglected presentation. J Neurol (2012) 259(1):4–21. 10.1007/s00415-011-6130-x 21743992

[3] Minor LB Solomon D Zinreich JS Zee DS. Sound- and/or pressure-induced vertigo due to bone dehiscence of the superior semicircular canal. Arch Otolaryngol Head Neck Surg (1998) 124(3):249–58. 10.1001/archotol.124.3.249 9525507

[4] Grieser BJ Kleiser L Obrist D. Identifying mechanisms behind the Tullio phenomenon: a computational study based on first principles. J Assoc Res Otolaryngol (2016) 17(2):103–18. 10.1007/s10162-016-0553-0 26883248 PMC4791416

[5] Ho ML Moonis G Halpin CF Curtin HD. Spectrum of third window abnormalities: semicircular canal dehiscence and beyond. AJNR Am J Neuroradiol (2017) 38(1):2–9. 10.3174/ajnr.A4922 27561833 PMC7963676

[6] Koo JW Hong SK Kim DK Kim JS Superior semicircular canal dehiscence syndrome by the superior petrosal sinus. J Neurol Neurosurg Psychiatry (2010) 81:465–7. 10.1136/jnnp.2008.155564 20176603

[7] Ward BK Carey JP Minor LB. Superior canal dehiscence syndrome: lessons from the first 20 years. Front Neurol (2017) 8:177. 10.3389/fneur.2017.00177 28503164 PMC5408023

[8] Cremer PD Minor LB Carey JP Della Santina CC. Eye movements in patients with superior canal dehiscence syndrome align with the abnormal canal. Neurology (2000) 55:1833–41. 10.1212/WNL.55.12.1833 11134382

[9] Gianoli JG Soileau JS Superior semicircular canal dehiscence: pathophysiology and surgical outcomes. Curr Otorhinolaryngol Rep (2017) 5:153–9. 10.1007/s40136-017-0156-2

[10] Gubbels SP Zhang Qi BS Lenkowski PW Hansen MR. Repair of posterior semicircular canal dehiscence from a high jugular bulb. Ann Otol Rhinol Laryngol (2013) 122(4):269–72. 10.1177/000348941312200409 23697325 PMC3972486

[11] Hain T Available from: http://dizziness-and-balance.com/research/hsn/Head%20Shaking%20Nystagmus.htm last modified July 4, 2017; http://dizziness-and-balance.com/disorders/unilat/fistula.html last modified October 29, 2017; and http://dizziness-and-balance.com/practice/valsalva.html last modified December 23, 2014.

[12] Davis RE. Diagnosis and management of perilymph fistula: the University of North Carolina approach. Am J Otol (1992) 13(1):85–9. 1598994

[13] Dieterich M Brandt T Fries W. Otolith function in man. Results from a case of otolith Tullio phenomenon. Brain (1989) 112(Pt 5):1377–92. 10.1093/brain/112.5.1377 2804618

[14] Cawthorne T The effect on hearing in man of removal of the membranous lateral semicircular canal. Acta Otolaryngol (1948) 36:145–9. 10.3109/00016484809122648

[15] Lempert J Physiology of hearing; what have we learned about it following fenestration surgery? AMA Arch Otolaryngol (1952) 56:101–13. 10.1001/archotol.1952.00710020120001 14943329

[16] Zhang LC Sha Y Dai CF. Another etiology for vertigo due to idiopathic lateral semicircular canal bony defect. Auris Nasus Larynx (2011) 38(3):402–5. 10.1016/j.anl.2010.11.003 21216120

[17] Huizinga E The reaction of Tullio and the fenestration operation. Laryngoscope (1952) 62(7):741–51. 10.1288/00005537-195207000-00007 14947028

