# Supplementary material for: Superior Semicircular Canal Ampullae Dehiscence As Part of the Spectrum of the Third Window Abnormalities: A Case Study
Source: Front Neurol. 2017 Dec 19;8:683. doi: 10.3389/fneur.2017.00683 (PMC5742101; doi:10.3389/fneur.2017.00683)
Supplement: Supplementary file 1 [file Presentation_1.pptx]

## Slide 1
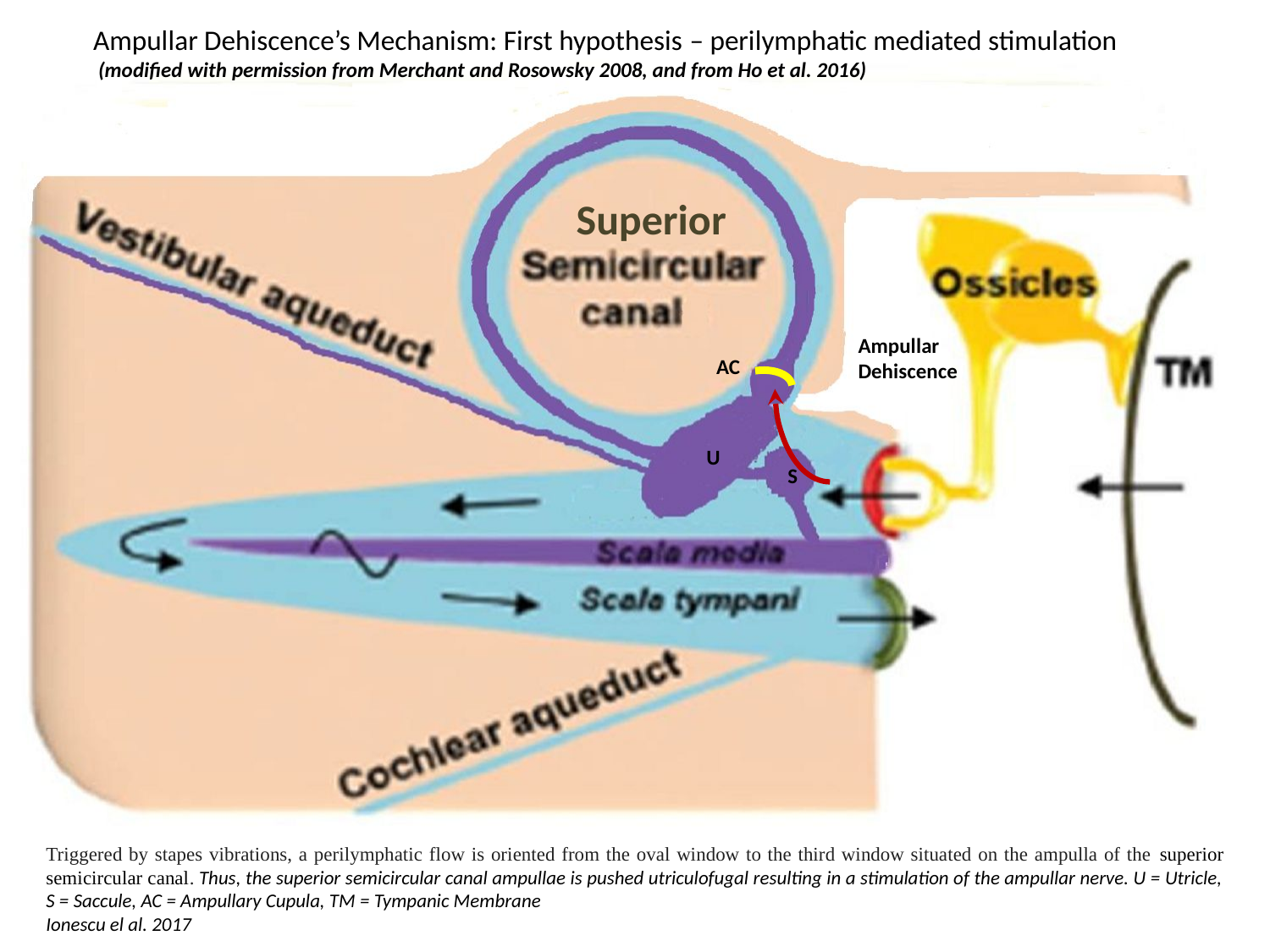

Superior
U
S
Ampullar Dehiscence’s Mechanism: First hypothesis – perilymphatic mediated stimulation
 (modified with permission from Merchant and Rosowsky 2008, and from Ho et al. 2016)
Ampullar
Dehiscence
AC
Triggered by stapes vibrations, a perilymphatic flow is oriented from the oval window to the third window situated on the ampulla of the superior semicircular canal. Thus, the superior semicircular canal ampullae is pushed utriculofugal resulting in a stimulation of the ampullar nerve. U = Utricle, S = Saccule, AC = Ampullary Cupula, TM = Tympanic Membrane
Ionescu el al. 2017

## Slide 2
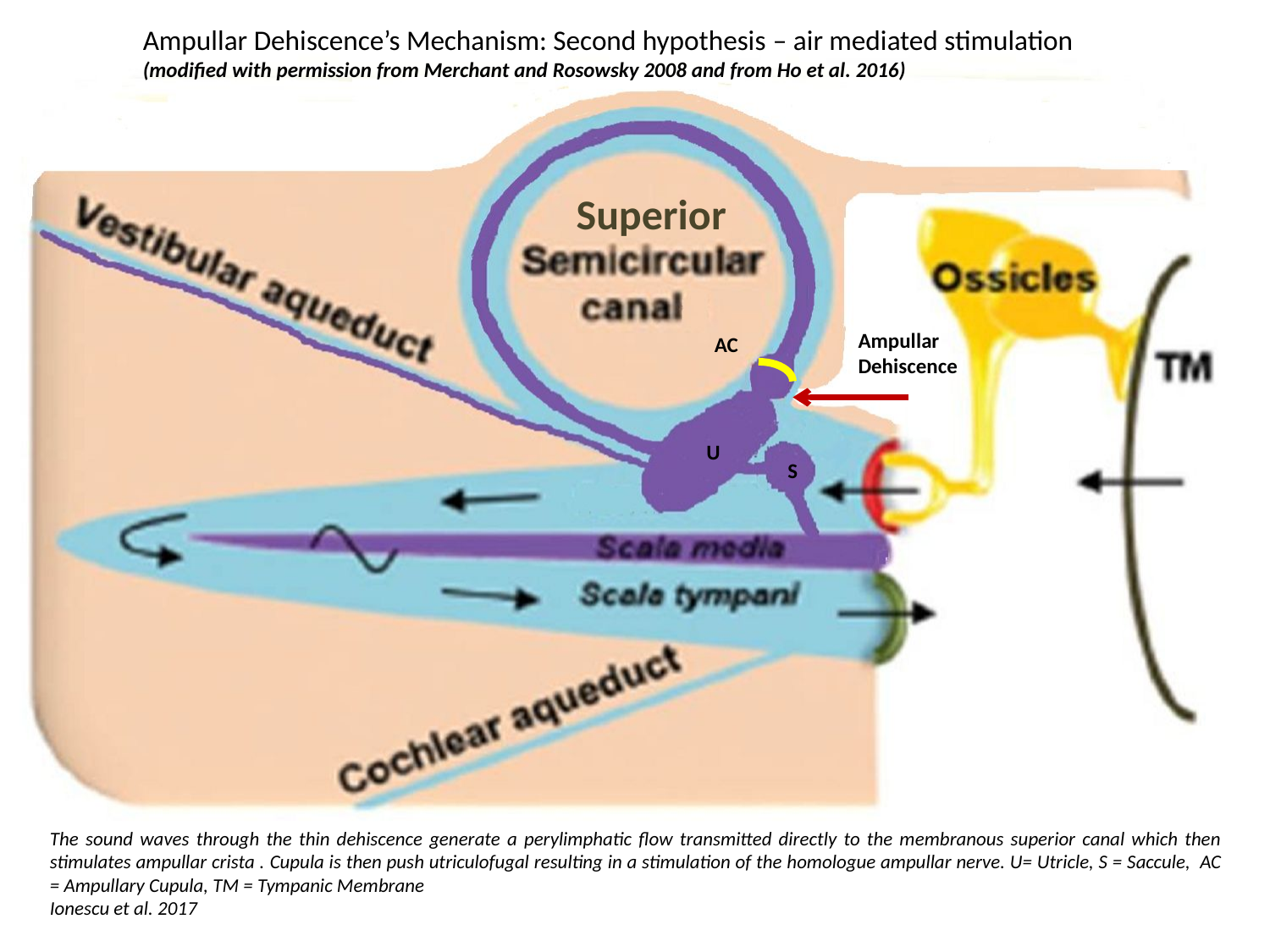

Superior
U
S
Ampullar
Dehiscence
Ampullar Dehiscence’s Mechanism: Second hypothesis – air mediated stimulation
(modified with permission from Merchant and Rosowsky 2008 and from Ho et al. 2016)
AC
The sound waves through the thin dehiscence generate a perylimphatic flow transmitted directly to the membranous superior canal which then stimulates ampullar crista . Cupula is then push utriculofugal resulting in a stimulation of the homologue ampullar nerve. U= Utricle, S = Saccule, AC = Ampullary Cupula, TM = Tympanic Membrane
Ionescu et al. 2017

## Slide 3
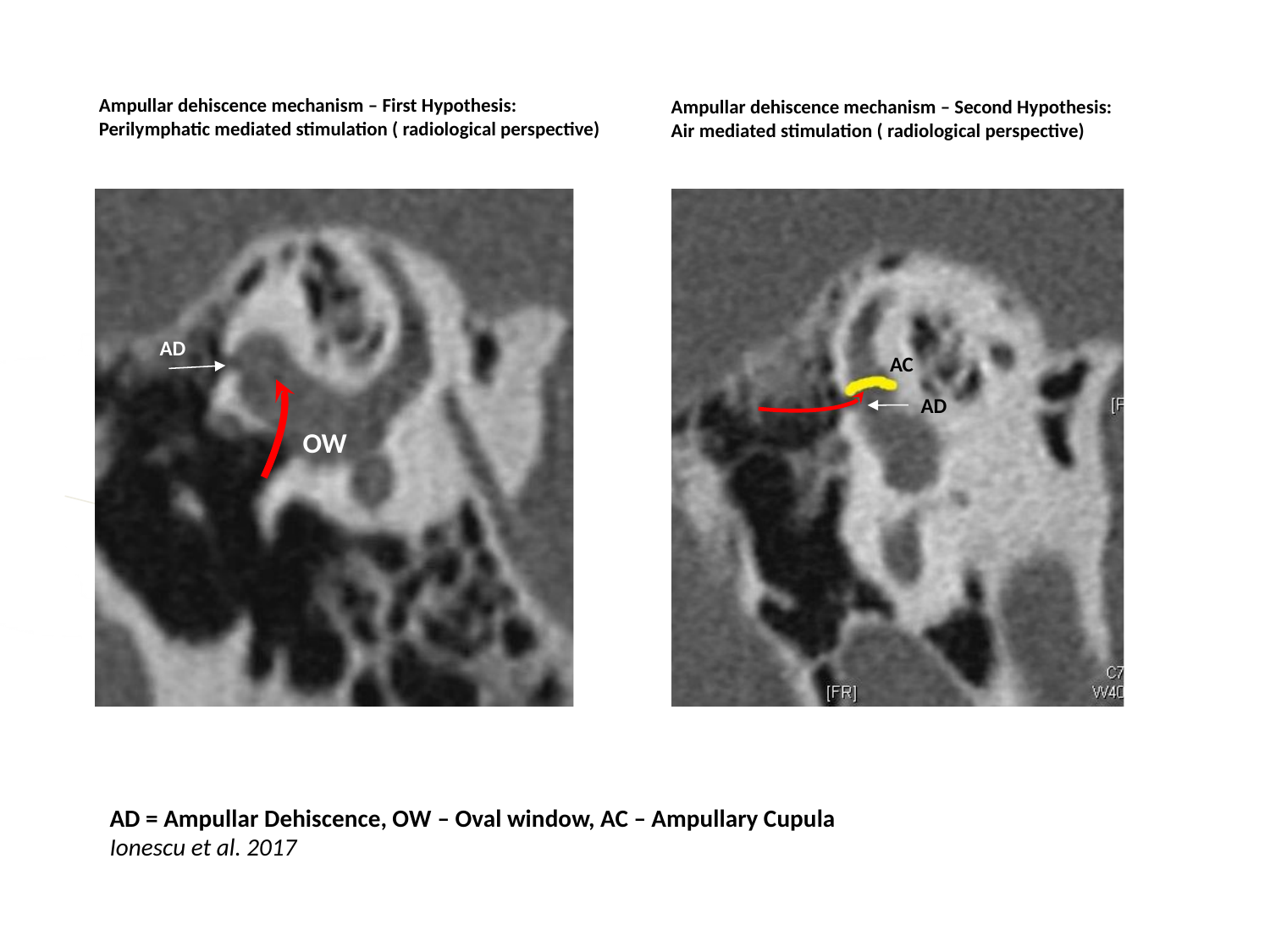

Ampullar dehiscence mechanism – First Hypothesis:
Perilymphatic mediated stimulation ( radiological perspective)
Ampullar dehiscence mechanism – Second Hypothesis:
Air mediated stimulation ( radiological perspective)
AD
AC
AD
~ 0,6mm
0,96mm
OW
AD = Ampullar Dehiscence, OW – Oval window, AC – Ampullary Cupula
Ionescu et al. 2017
